# A web-based database server using 43,710 public RNA-seq samples for the analysis of gene expression and alternative splicing in livestock animals

**DOI:** 10.1186/s12864-022-08881-2

**Published:** 2022-10-17

**Authors:** Jinding Liu, Kun Lang, Suxu Tan, Wencai Jie, Yihua Zhu, Shiqing Huang, Wen Huang

**Affiliations:** 1 Bioinformatics Center, Academy for Advanced Interdisciplinary Studies, Nanjing Agricultural University, Nanjing, 210095 Jiangsu China; 2 Department of Animal Science, Michigan State University, East Lansing, MI 48824 USA; 3 College of Information Management, Nanjing Agricultural University, Nanjing, 210095 Jiangsu China; 4 State Key Laboratory of Pharmaceutical Biotechnology, School of Life Sciences, Nanjing University, Nanjing, 210023 Jiangsu China

**Keywords:** Livestock, Gene expression, Alternative splicing, RNA-seq, Database

## Abstract

**Background:**

Livestock animals is of great significance in agricultural production. However, the role of specific gene expression, especially alternative splicing in determining phenotype, is not well understood. The livestock research community needs a gene expression and alternative splicing database contributing to livestock genetic improvement.

**Description:**

We report the construction of LivestockExp (https://bioinfo.njau.edu.cn/livestockExp), a web-based database server for the exploration of gene expression and alternative splicing using 43,710 uniformly processed RNA-seq samples from livestock animals and several relative species across six orders. The database is equipped with basic querying functions and multiple online analysis modules including differential/specific expression analysis, co-expression network analysis, and cross-species gene expression conservation analysis. In addition to the re-analysis of public datasets, users can upload personal datasets to perform co-analysis with public datasets. The database also offers a wide range of visualization tools and diverse links to external databases enabling users to efficiently explore the results and to gain additional insights.

**Conclusion:**

LivestockExp covers by far the largest number of livestock animal species and RNA-seq samples and provides a valuable data resource and analysis platform for the convenient utilization of public RNA-seq datasets.

**Supplementary Information:**

The online version contains supplementary material available at 10.1186/s12864-022-08881-2.

## Background

To generate hypotheses regarding specific genes, biologists often perform transcriptomic profiling involving gene expression and alternative splicing at the whole genome level. High-throughput RNA sequencing (RNA-seq) is a rapid and cost-effective way to obtain large amounts transcriptome data from diverse organisms and tissue types [1]. Over the last decade, this method has become a routine but indispensable approach for systematically characterizing the transcriptome across diverse developmental stages and other experimental conditions. It has produced a massive amount of data deposited in public repositories. For example, the Sequence Read Archive (SRA) database at NCBI is the largest publicly available repository of high throughput sequencing data with more than 3.3 million RNA-seq samples.

Researchers can obtain more new insights by retrospective analyses based on the collection of RNA-seq data across studies [2, 3]. For a typical experimental biologist facing such large volumes of data, the utilization of these data has been difficult because of the vast extent of the data and the requirement for specialized computational resources and bioinformatics skills. A key step towards the better utilization of diverse public RNA-seq data involves the uniform processing of sequence data. For humans and mice, a total of 750,000 RNA-seq samples have been uniformly processed to enable re-analyses by collection of RNA-seq samples across different studies [3]. MetazExp developed by our group has uniformly processed 53,615 publicly RNA-seq samples and offers multiple retrieval and analysis functions for users to explore metazoan gene expression and alternative splicing [4]. Livestock animals are of extremely important economic value in agriculture, and so some databases have been developed for their genetic improvement. For example, GGVD was developed to access dense and broadly representative goat whole-genome variation data [5]. However, the specific roles of gene expression and alternative splicing in determining phenotypic variation is not well understood. The community needs a gene expression and alternative splicing database contributing to livestock genetic improvement.

**Fig. 1:**
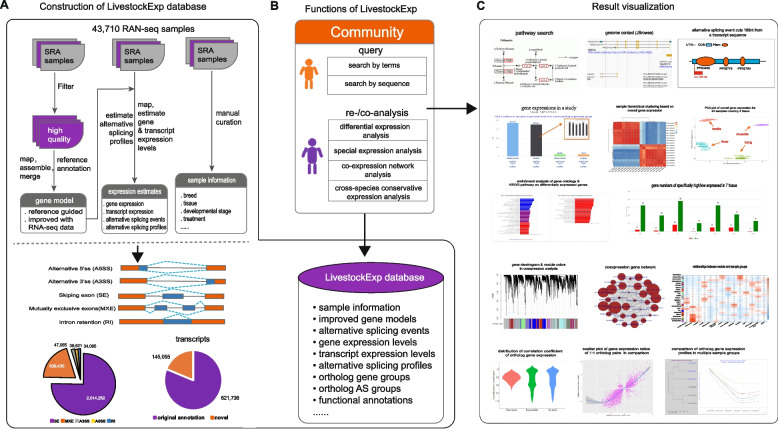
Overview of LivestockExp. **A** Database construction and content. **B** LivestockExp provides users with tools to query the database and to re- and co-analyze public data. **C** Result visualization returned from LivestockExp. The KEGG pathway was reproduced with written permission from the KEGG

Here, we present LivestockExp (Fig. 1), a web-based gene expression and alternative splicing platform that builds upon 43,710 publicly available RNA-seq samples of the most livestock animals and several species across six orders. LivestockExp extends our previous work focusing on a database server only for alternative splicing, ASlive [6]. It has several important features that distinguishes it from other databases. First, it covers nearly all livestock animals and the largest number of livestock RNA-seq samples. Second, in addition to gene expression, it also covers gene alternative splicing that plays a vital role in animal development and growth. Third, the online analysis modules allow users to perform re-analysis of public datasets and/or co-analysis with their own datasets. Fourth, it supports cross-species gene expression conservation analysis that is unavailable in other similar databases.

## Construction and content

### RNA-seq data and sample curation

LivestockExp includes most livestock animals and serval relative species covering six orders (Table 1). Their reference genomes and annotations were all collected from the Ensembl database [7]. We only considered RNA-seq data generated by the Illumina platform in the SRA database. A total of 43,710 RNA-seq samples from 2125 studies containing 242.485 T-bases were gathered for construction of the database (Table 1, Table S1). The three species comprising most of the data were cow, pig, and chicken, having 10,774, 9936, and 9092 samples and accounting for 24, 22, and 20%, respectively.

**Table 1:** Summary of genomes and RNA-seq samples collected in LivestockExp

Order	Scientific name	Common name	Studies	Samples	Volume (GB)
Artiodactyla	*Bos taurus*	Cow	384	10,774	45,669
	*Bos grunniens*	Domestic yak	29	246	2710
	*Ovis aries*	Sheep	184	3749	27,679
	*Capra hircus*	Goat	119	1620	12,915
	*Sus scrofa*	Pig	517	9936	57,022
Perissodactyla	*Equus caballus*	Horse	83	2025	9667
	*Equus asinus*	Donkey	13	75	558
Carnivora	*Canis lupus familiaris*	Dog	146	2899	16,010
	*Felis catus*	Cat	21	251	2021
Lagomorpha	*Oryctolagus cuniculus*	Rabbit	63	1344	7972
Galliformes	*Gallus gallus*	Chicken	474	9092	51,651
	*Meleagris gallopavo*	Turkey	12	890	2082
Anseriformes	*Anas platyrhynchos*	Mallard	61	596	4509
	*Anser cygnoides*	Swan goose	19	213	2020
Sum	–	–	2125	43,710	242,485

To allow the convenient re-analysis of the collected samples, we manually curated sample attributes focusing on breed, genotype, tissue, developmental stage, and treatment (experimental conditions) based on the description of RNA-seq sample offered by submitters or related publications.

### Gene model improvement

The gene annotations of livestock animals were largely incomplete, unlike those of model species. For example, although human and cattle have similar genome sizes, there are 5.1 alternative splicing isoforms per human gene versus 1.78 per cattle gene [7]. We adopted a procedure similar to that described previously [4] to improve gene model annotation and obtained a uniform annotation for each species as the final reference in LivestockExp. Briefly, fastp v 0.23.0 [8] was used for trimming and filtering raw reads, and Hisat v2.1.0 [9] was used for alignment to evaluate the data quality. The RNA-seq data with at least 80% unique mapping rate, at least 100 bp in read length, and at least a certain sequencing depth were used to improve gene model annotation. The requirement of sequencing depth varied depending on the availability in corresponding species and genome size. For example, at least 6G bases needed to be mapped in pig and cattle to qualify as a dataset that was used for gene model construction. The read alignments on the genome were assembled into reference guided gene models by using StringTie2 v2.1.4 [10]. The resulting transcriptome in GTF format were compared iteratively with the merged transcriptome using cuffcompare [1]. We only retained novel multi-exonic transcripts at least 200 bp long with at least 2x coverage per transcript and 1x per exon for all exons. Finally, novel transcripts were further filtered to retain that occur in at least 3 samples and account for at least 50% samples of any tissue or at least 1/3 of all samples.

After this process, the gene models contained more splicing junctions, exons, and alternative transcripts. Splice junctions and exons on average increased by 5.1 and 1.36%, respectively (Table S2). The average proportion of multi-exonic genes with multiple isoforms increased from 40.27 to 55.77% (Table S2). The average number of alternative splicing isoforms per multi-exonic gene increased from 1.8 to 2.35.

### Gene function annotation

To support retrieval by functional terms and functional enrichment analysis, diverse gene function annotations were integrated in LivestockExp, including protein domain, gene ontology terms, and KEGG pathways. The gene ontology terms were obtained directly from the reference annotations in Ensembl. Interproscan [11] was used to annotate protein families and conservative protein domains. Protein sequences were submitted to KAAS (KEGG automatic annotation server) to obtain KO (KEGG Orthology) assignments and corresponding KEGG pathways [12].

### Estimation of gene expression

StringTie was used to estimate gene/transcript expression levels based on alignments from Hisat2. LivestockExp included two gene expression metrics including TPM (Transcripts Per Million) and FPKM (Fragments Per Kilobase of gene per Million mapped fragments). To support differential expression analysis, raw read count numbers were also obtained using the Python script prepDE.py release accompanying StringTie2.

### Identification and quantification of alternative splicing

LivestockExp gathered five classic alternative splicing events including skipped exon (SE), mutually exclusive exons (MXE), alternative 5′ splice sites (A5SS), alternative 3′ splice sites (A3SS) and retained intron (RI) (Fig. 1A). rMATS v 4.0.2 [13] was used to call alternative splicing events. In total, 2,674,353 alternative splicing events were identified from 43,710 samples. The most abundant alternative splicing type is SE, accounting for 61.29% in each sample on average. In the alternative splicing events occurring in each sample, an average of 67.55% was derived from GTF annotation, with 32.45% being novel and discovered from read alignments on exons.

Alternative splicing profiles are quantified by raw read count numbers and Percent Spliced-In (PSI). The PSI value is defined as the percentage of the exon inclusion transcripts [13]. The PSI values are calculated by JCEC and JC methods, respectively. In the former, event counts include both reads that span junctions (Junction Counts) and reads that do not cross an exon boundary (Exon Counts), whereas in the latter, event counts include only reads that span junctions (Junction Counts).

### Identification of orthologous gene and alternative splicing groups

First, we identified orthologous gene groups based on the longest protein sequences of genes by using orthofinder [14]. The protein sequences in ortholog groups were then aligned globally with mafft [15] and converted to codon alignments with pal2nal [16]. Finally, we calculated the new coordinates of exons in transcripts corresponding to the longest protein based on the codon alignments. Alternative splicing events with the same coordinates based on codon alignments were classified into an ortholog group.

In total, 18,911 orthologous gene groups were identified from 14 species. Based on these orthologous genes, we identified 265,994 putative orthologous alternative splicing groups that contain 1,230,616 events and covered at least two species.

### Online analysis modules

Four online modules are implemented in LivestockExp, including differential expression, specific expression, co-expression network, and cross-species gene expression conservation analysis. DESeq2 [17] and edgeR [18] are employed to detect differentially expressed genes. Three statistical models, MATS_LTR, rMATS_unpaired and rMATS_paired, implemented in rMATS [13] are used to discover differentially alternative splicing events. The hypergeometric test and Gene Set Enrichment Analysis (GSEA) implemented in the R package ClusterProfiler [19] are integrated into LivestockExp for gene ontology (GO) and KEGG pathway enrichment analysis [20]. WGCNA [21] is used for weighted gene co-expression network analysis. In the cross-species gene expression conservation analysis module, phylip v3.696 [22] is employed to build a molecular phylogenetic tree. The gene expression levels are normalized to compare orthologous gene expression profiles. The normalized expression values are the log2-transformed ratios of the expression of a gene in a sample divided by the trimmed mean expression level.

To enhance analysis performance, when possible (study not completely confounded with experimental conditions), the R package SVA [23] was used to adjust the batch effect attributable to studies.

### Database implementation

All data are stored and managed with MySQL (https://dev.mysql.com; a free relational database management system). PHP v5.4.16 (https://www.php.net, a server side scripting language embedded in HTML) and R v4.0.2 (https://cran.r-project.org; a programming language for statistical analysis) language are used for sever-side scripting, as are JavaScript and JQuery (https://jquery.com; a fast, small, and feature-rich JavaScript library) as the front-end interface. Furthermore, data visualization is built by HighCharts (https://www.highcharts.com; a JavaScript plug-in to create interactive charts) and DataTables (https://datatables.net; a plug-in for the jQuery JavaScript library to render HTML tables). JBrowse [24] (https://jbrowse.org/jbrowse1.html; a fast and scalable genome browser built completely with JavaScript and HTML5) is also embedded in LivestockExp for users to explore gene, transcript, and alternative splicing structures in a genomic context.

## Utility and discussion

LivestockExp is hosted at https://bioinfo.njau.edu.cn/livestockExp. It has a basic function for querying gene expression and alternative splicing profiles from the database. It is also equipped with multiple online modules to re-analyze public data and/or to co-analyze a user’s own data.

### Querying and result visualization

**Fig. 2:**
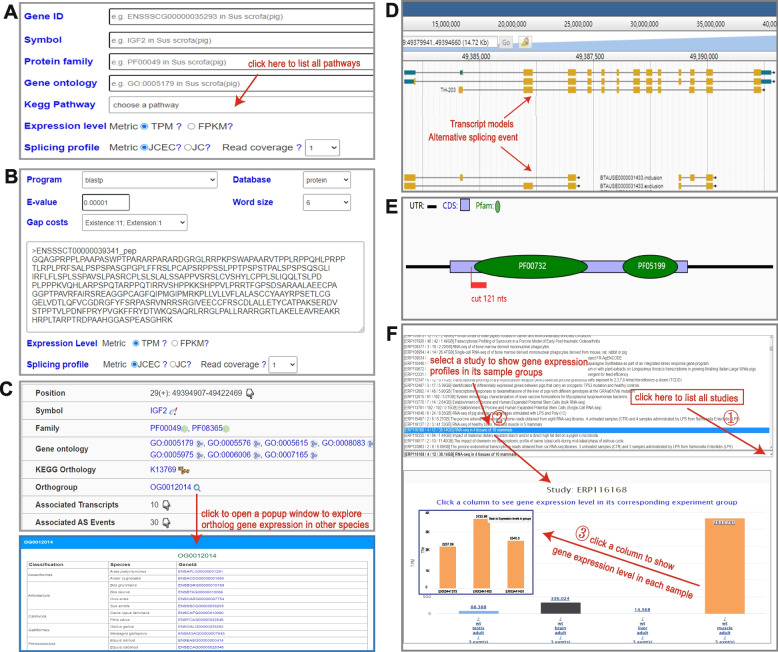
Retrieval functions and result visualization in LivestockExp. **A** Genes can be queried by ID, protein family, GO terms, or KEGG pathway on the search page. **B** Alternatively, genes can be searched by nucleotide or protein sequence on the blast page. **C** The basic features of a retrieved gene are listed on the top of the gene page. By clicking on the ortholog group ID, users can open a popup window to explore ortholog gene expression in other species. **D** JBrowse showing the associated transcript models and alternative splicing events within a gene locus. **E** Impact of alternative splicing on a transcript with protein domains. This alternative splicing removes 121nts from the transcript and leads to a destructive effect on the PF00732 protein domain. **F** A list box showing all studies and an interactive bar chart showing gene expression levels in sample groups from the ERP116168 study. The bar chart can be clicked to expand and to visualize the expression level in each sample

For each species, there are two ways to query gene expression and alternative splicing. In the search page, users can query the database by gene ID, symbol, and functional terms such as Pfam and GO annotations or by gene list in pathways (Fig. 2A). Alternatively, users can search orthologous genes by sequence similarity in the blast page (Fig. 2B). In these two pages, users can specify metrics to show gene expression levels and alternative splicing profiles.

Basic features and expression levels of a retrieved gene are organized within the gene page. First, functional annotation terms of a gene are shown on the top with links for users to explore details in the corresponding external databases (Fig. 2C). Moreover, users can conveniently explore orthologs in other species by clicking ortholog group ID (Fig. 2C). JBrowse is embedded for users to explore genes, transcripts, and alternative splicing events in a genomic context (Fig. 2D). Next, a structure graph is used to display positions of the Pfam domain in the transcript sequence and the impact of alternative splicing on the transcript (Fig. 2E). Finally, a list box shows all of the collected studies, and a hierarchical and interactive bar chart shows the gene expression levels in a selected study (Fig. 2F). A bar in the top-level chart represents a sample group. It can be further expanded to show the diversity of expression within the same group.

Furthermore, a gene page presents two tables listing the associated transcripts and alternative splicing events of the gene with links for users to open the transcript page or alternative splicing page to show corresponding details. Information on the transcript and alternative splicing pages has an organizational structure similar to that on the gene page.

### Differential and specific expression analysis

**Fig. 3:**
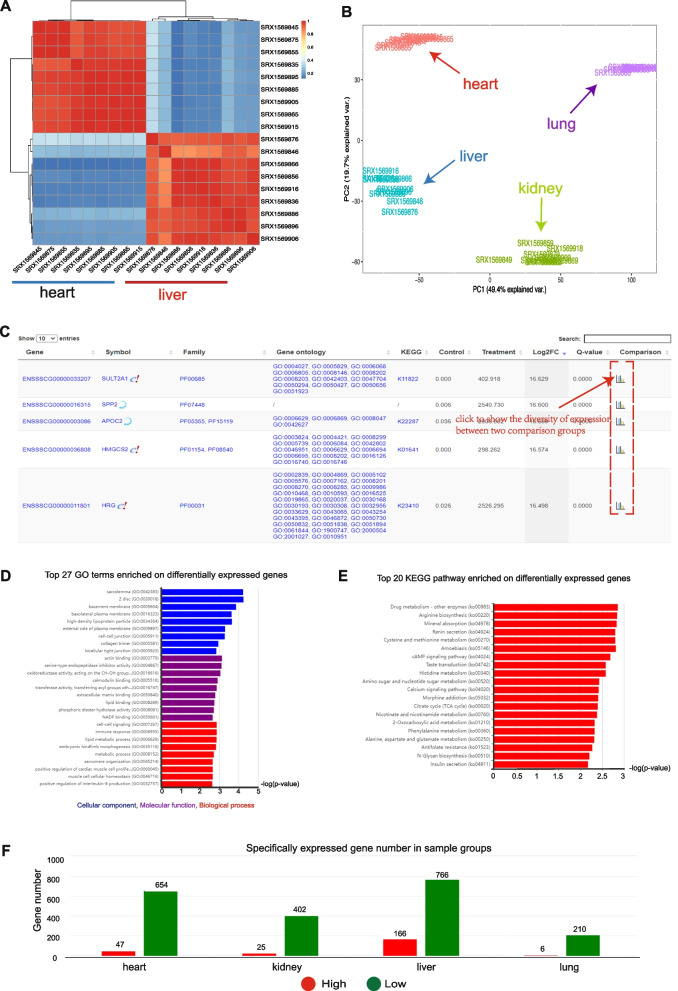
Results returned from differential and specific analyses. **A** Heatmap showing hierarchical clustering of nine heart and nine liver samples based on overall gene expression. **B** PCA plot of overall gene expression for 36 samples covering heart, liver, kidney, and lung tissues. **C** Differential expression analysis result is displayed in an interactive table with links for users to view the diversity of gene expression between two comparison groups. **D** Enriched gene ontology categories for differentially expressed genes between heart and liver samples. The blue, purple, and red bars respectively represent enriched cellular component, molecular function, and biological process terms. **E** Enriched KEGG pathways for differentially expressed genes between heart and liver samples. **F** Summary of specifically high- and low-expressed genes in heart, liver, kidney, and lung sample groups. Red and green bars represent specifically high- and low-expressed genes, respectively

In differential and specific expression analysis modules, users can flexibly customize sample groups to explore gene expression changes. LivestockExp offers heat maps (Fig. 3A) and principal component analysis (PCA) graphs (Fig. 3B) illustrating clustering of samples based on global gene expression or alternative splicing. Two interactive tables show differentially/specifically expressed genes or differentially/specially spliced genes (Fig. 3C). In addition, GO and KEGG pathway enrichment analysis can also be performed on the differentially/specifically expressed and spliced genes (Fig. 3D, E). The numbers of specifically expressed genes and specifically alternative splicing events are summarized by groups in a bar chart (Fig. 3F).

### Co-expression network analysis

**Fig. 4:**
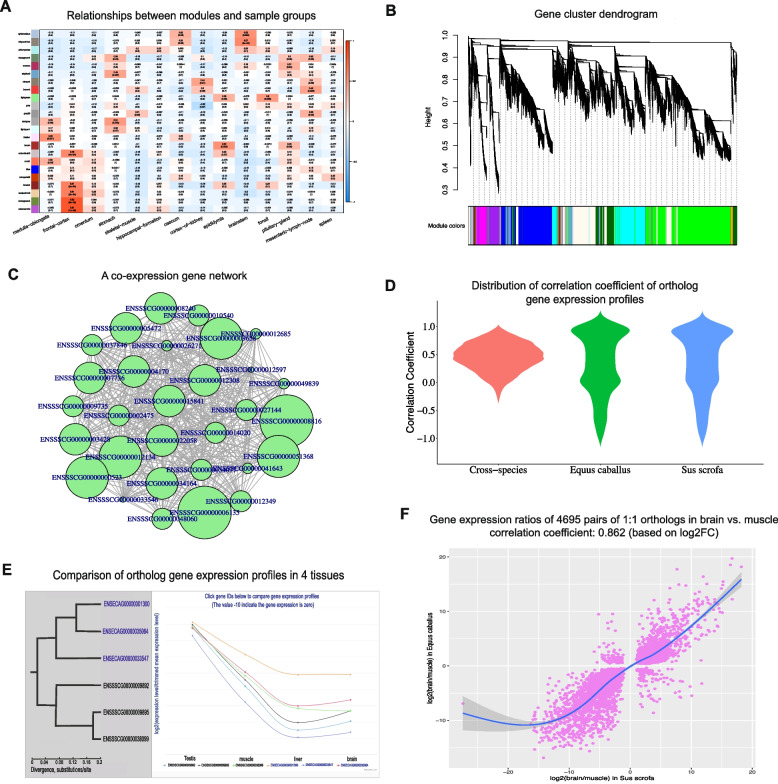
Results returned from co-expression network and cross-species expression conservation analyses. **A** A heat map showing relationships between modules and sample groups. The correlation and significance within a cell measures the degree of relationships between the corresponding module and sample group. **B** A gene dendrogram of clustered modules. **C** A network representing a gene module. Sizes of nodes correspond to degrees of connectivity (number of connections). The largest nodes tend to be hub genes. **D** Violin graphs showing distribution of correlation coefficient of ortholog gene expression profiles across sample groups. **E** Molecular phylogenetic tree of ortholog genes and their expression profile comparison across four tissues. **F** Scatter plot of gene expression ratios of 4695 pairs of 1:1 orthologs (horse-pig) in brain vs. muscle. Up- or downregulation of a gene in horse brain is highly predictive of up- or downregulation of its pig ortholog in brain

Weighted Gene Co-expression Network Analysis (WGCNA) is utilized to partition the gene co-expression networks into clusters or modules. These networks are then correlated with sample groups divided by breed, tissue, developmental stage, or treatment conditions. With this analysis function, LivestockExp offers diverse graphs or tables to display analysis results, such as a heat map showing relationships between gene modules and sample groups (Fig. 4A), a gene dendrogram of clustered dissimilarity (Fig. 4B), and network graphs representing gene modules in which the larger nodes tend to be hub genes (Fig. 4C). As in the differential expression analysis module, GO and KEGG pathway enrichment analysis can also be performed on all gene modules in order to learn the function of the identified gene modules.

### Cross-species gene expression conservation analysis

With the cross-species gene expression conservation analysis module, users can rapidly learn the consistency of ortholog gene expression, intra- and inter-species, covering multiple sample groups (Fig. 4D). For an ortholog gene group of interest, users can open a new page to build a molecular phylogenetic tree and dynamically compare gene expression profiles covering multiple sample groups (Fig. 4E). For example, users can compare differential expression between tissues across species (Fig. 4F).

## A case study to explore the regulation of gene expression and alternative splicing in satellite cells of pig skeletal muscle

**Fig. 5:**
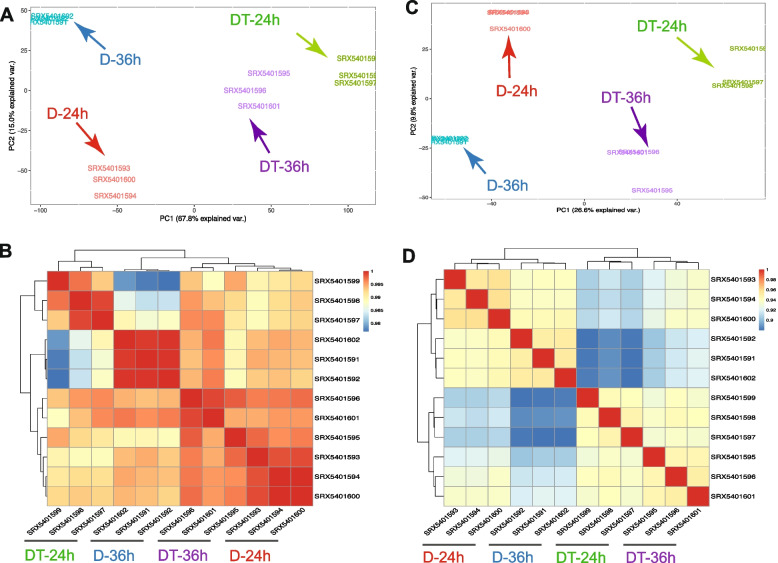
Case study to analyze gene expression and alternative splicing in satellite cells of pig skeletal muscle (PSCs) by using LivestockExp. **A** PCA plot of overall gene expression for samples covering four group of PSCs. D-24 h and D-36 h indicate induced differentiation for 24 and 36 hours, respectively, in PSCs. DT-24 h and DT-36 h indicate the corresponding PSCs after transfected with antisense oligonucleotide of TCONS_00323213. **B** Hierarchical clustering based on overall gene expression. **C** Same samples but plotted for PCA of overall alternative splicing. **D** Hierarchical clustering based on alternative splicing (PSI)

To illustrate the power of LivestockExp, we present here a case study exploring gene expression and alternative splicing in the satellite cells of pig skeletal muscle (PSCs) based on a published SRA bioproject with accession SRP186451. This analysis includes four PSC sample groups with three replicates for each group. In two out of the four groups, differentiation was induced for 24 hours (D-24 h) and 36 hours (D-36 h), respectively. The other two groups were transfected with an antisense oligonucleotide of a long non-coding RNA (TCONS_00323213), and differentiation was induced, respectively, for 24 hours (DT-24 h) and 36 hours (DT-36 h). The 12 samples were well clustered into four groups, respectively corresponding to four experimental conditions based on both gene expression (Fig. 5A and B) and alternative splicing profiles (Fig. 5C and D) suggesting that the public RNA-seq datasets have high applicability.

DESeq2 and MATS_LRT with default parameters were respectively used to discover differentially expressed genes and alternative splicing events between D-24 h and D-36 h. In addition to 2439 differentially expressed genes, we found 1163 differential alternative splicing events. Hypergeometric tests with default parameters were used in an enrichment analysis. We found that the differentially expressed genes were enriched in 42 GO terms and in 21 pathways, whereas the alternative splicing genes were enriched in 10 GO terms and in two pathways. Only two GO terms were shared by the differentially expressed and alternative splicing genes (Table S3–6). Alternative splicing generates complex proteomes and plays a vital role in mammalian development and growth [25]. The results of this analysis thus revealed that the differentiation of PSCs was also regulated by a large number of alternative splicing events.

Long non-coding RNA ubiquitously participates in the dynamic regulation process of myogenesis [26]. With the same methods and parameters, we found both gene expression and alternative splicing profiles changed dramatically after PSCs were transfected with the antisense oligonucleotide of TCONS_00323213. LivestockExp reported 18,436 differentially expressed genes and 2036 differential alternative splicing events in DT-24 h, and 14,635 differentially expressed genes and 2193 differential alternative splicing events in DT-36 h. In the two comparisons, the same GO terms and pathways were not enriched in either the differentially expressed genes or the alternative splicing genes (Table S7–14). The analyses indicated that both gene expression and alternative splicing profiles were regulated by the long non-coding RNA TCONS_00323213. These differentially expressed and alternative splicing genes and the related analyses provide meaningful reference points involved in the development of pig skeletal muscle.

Nucleotide sequence read databases, such as the SRAdb database, hold a tremendous amount of high throughput sequencing data covering multiple life domains, but they are difficult to access and re-analyse rapidly because only raw sequence reads are available. To simplify access and re-analysis, LivestockExp is specifically designed for livestock and has some special features. Some of these features are individual or are common to other gene expression databases. We discuss these features in turn.

First, LivestockExp is the largest and most comprehensive gene expression analysis platform covering 43,710 RNA-seq samples across nearly all types of livestock. In addition to gene/transcript expression profiles, it includes gene alternative splicing profiles that tend to be ignored by many databases but that are an important aspect affecting gene functions by altering protein products [6, 27].

Second, we have improved gene models and thus provide a foundation for the identification of alternative splicing events. The discovery of alternative splicing events depends on read alignments on exons [13]. Incomplete annotations affect the downstream identification of alternative splicing events. Many databases ignore gene model improvement [28–30]. This may have little impact on model species but a large impact on non-model species.

Third, our uniform data processing feature contributes to the re-analysis and co-analysis of public RNA-seq datasets. Retrospective analyses of previously published RNA-seq data can provide new insights for researchers [31]. However, many post hoc studies depend on large data sets that are inter-operable with the data analysis pipeline, whereby gene expression data is provided in a processed form. Several attempts have been made to create unified resources enabling the cross-study analysis of RNA-seq data [2, 4, 28]. In LivestockExp, the integrated gene expression and alternative splicing profiles are all processed by uniform methods.

Fourth, sample curation increases the availability of public RNA-seq datasets. In biology, researchers often perform analyses on several experimental groups in to construct hypotheses [17, 18]. The description of RNA-seq samples in the SRA database are not controlled [32]. To reuse these public RNA-seq datasets in a satisfactory manner, we manually curate sample attributes focusing on breed, genotype, tissue, developmental stage, and treatment conditions. These attributes are of common interest for researchers. With the specific descriptive structures, the retrieved gene expression profiles can be divided into various groups, and the differences between/among the different experimental groups can be easily viewed. In addition, when undertaking the re- and co-analysis of public RNA-seq datasets, users can flexibly customize groups of interest to them according to the curated sample attributes.

Finally, cross-species gene expression conservation analysis is a unique function. This feature is of great significance for comprehensively understanding a gene in depth [33]. Almost all gene expression databases lack this function. LivestockExp allows users to explore the conservation of cross-species expression by using this module, which involves the setting of multiple experimental groups. Moreover, users can compare expression profiles of ortholog genes and, concomitantly, investigate their evolutionary relationship within a molecular phylogenetic tree.

## Conclusions

In this work, we have constructed LivestockExp database including improved gene models, alternative splicing events and gene expression and alternative splicing profiles in 43, 710 livestock RNA-seq samples. In addition to common search functions, it allows users to perform multiple online analysis functions to explore complex datasets by customizing sample groups. Thus, it is a valuable data resource and analysis platform for the livestock research community to conveniently utilize public RNA-seq datasets.

## Supplementary Information

**Additional file 1: Table S1.** All SRA accessions used in this study. **Table S2.** Improvement of gene annotation. **Table S3.** Enriched GO terms on differentially expressed genes (D-36h vs D-24h). **Table S4.** Enriched GO terms on differentially alternative splicing genes (D-36h vs D-24h). **Table S5.** Enriched pathways on differentially expressed genes (D-36h vs D-24h). **Table S6.** Enriched pathways on differentially alternative splicing genes (D-36h vs D-24h). **Table S7.** Enriched GO terms on differentially expressed genes (DT-24h vs D-24h). **Table S8.** Enriched GO terms on differentially alternative splicing genes (DT-24h vs D-24h). **Table S9.** Enriched pathways on differentially expressed genes (DT-24h vs D-24h). **Table S10.** Enriched pathways on differentially alternative splicing genes (DT-24h vs D-24h). **Table S11.** Enriched GO terms on differentially expressed genes (DT-36h vs D-36h). **Table S12.** Enriched GO terms on differentially alternative splicing genes (DT-36h vs D-36h). **Table S13.** Enriched pathways on differentially expressed genes (DT-36h vs D-36h). **Table S14.** Enriched pathways on differentially alternative splicing genes (DT-36h vs D-36h).

## Data Availability

Data pertinent to the database construction were downloaded from the Sequence Read Archive (SRA) and Ensembl as described above. The datasets generated during the construction of LivestockExp are available at: https://bioinfo.njau.edu.cn/livestockExp. All the SRA accession numbers used are listed in Table S1.

## Abbreviations

SRA: Sequence Read Archive
KASS: KEGG automatic annotation server
KO: KEGG Orthology
TPM: Transcripts per million
FPKM: Fragments per kilo base of gene per million mapped fragments
SE: Skipped exon
MXE: Mutually exclusive exon
A5SS: Alternative 5′ splice site
A3SS: Alternative 3′ splice site
RI: Retained intron
PSI: Percent spliced in
JCEC: Junction counts and exon counts
JC: Junction counts
GSEA: Gene set enrichment analysis
GO: Gene ontology
WGCNA: Weighted gene co-expression network analysis
PCA: Principal component analysis
